# Swarm Intelligence and Its Applications

**DOI:** 10.1155/2013/528069

**Published:** 2013-10-10

**Authors:** Yudong Zhang, Praveen Agarwal, Vishal Bhatnagar, Saeed Balochian, Jie Yan

**Affiliations:** ^1^School of Computer Science and Technology, Nanjing Normal University, Nanjing, Jiangsu 210023, China; ^2^Department of Mathematics, Anand International College of Engineering, Near Kanota, Agra Road, Jaipur 303012, India; ^3^Ambedkar Institute of Advanced Communication Technologies and Research, New Delhi, India; ^4^Department of Electrical Engineering, Islamic Azad University, Gonabad Branch, Gonabad, Iran; ^5^Suzhou University, Suzhou, Jiangsu, China

Swarm intelligence (SI) is the collective behavior of decentralized, self-organized systems, natural or artificial. SI systems are typically made up of a population of simple agents interacting locally with one another and with their environment. The inspiration often comes from nature, especially biological systems. The agents follow very simple rules, and although there is no centralized control structure dictating how individual agents should behave, local, and to a certain degree random, interactions between such agents lead to the emergence of “intelligent” global behavior, unknown to the individual agents. Natural examples of SI include ant colonies, bird flocking, animal herding, bacterial growth, and fish schooling.

Research in SI started in the late 1980s. Besides the applications to conventional optimization problems, SI can be employed in library materials acquisition, communications, medical dataset classification, dynamic control, heating system planning, moving objects tracking, and prediction. Indeed, SI can be applied to a variety of fields in fundamental research, engineering, industries, and social sciences.

The main objective of this special issue is to provide the readers with a collection of high quality research articles that address the broad challenges in application aspects of swarm intelligence and reflect the emerging trends in state-of-the-art algorithms. 

The special issue received 42 high quality submissions from different countries all over the world. All submitted papers followed the same standard (peer-reviewed by at least three independent reviewers) as applied to regular submissions to “this journal”. Due to the limited space, 15 papers were finally included. The primary guideline was to demonstrate the wide scope of SI algorithms and applications in various aspects. Besides, mathematically oriented papers with promising potential in practical problems were also included.

The paper authored by Y.-L. Wu et al. (National Chiao Tung University and Ming Chuan University) presents an integer programming model of the studied problem by considering how to select materials in order to maximize the average preference and the budget execution rate under some practical restrictions including departmental budget and limitation of the number of materials in each category and each language. They propose a discrete particle swarm optimization (DPSO) with scout particles, design an initialization algorithm and a penalty function to cope with the constraints, and employ the scout particles to enhance the exploration within the solution space. 

In the paper by Z. Yin et al. (Harbin Institute of Technology), they propose an efficient multiuser detector based on a suboptimal code mapping multiuser detector and artificial bee colony algorithm (SCM-ABC-MUD) and implement the proposed algorithm in direct-sequence ultrawideband (DS-UWB) systems under the additive white Gaussian noise (AWGN) channel.

M. S. Uzer et al. (Selçuk University) offer a hybrid approach that uses the artificial bee colony (ABC) algorithm for feature selection and support vector machines for classification. For the diagnosis of hepatitis, liver disorders, and diabetes datasets from the UCI database, the proposed system reached classification accuracies of 94.92%, 74.81%, and 79.29%, respectively.

Another paper is by M. Karakose (Fırat University) and U. Cigdem (Gaziosmanpaşa University). It proposes a new approach for improvement of DNA computing with adaptive parameters towards the desired goal using quantum-behaved particle swarm optimization (QPSO). Experimental results obtained with MATLAB and FPGA demonstrate ability to provide effective optimization, considerable convergence speed, and high accuracy according to DNA computing algorithm.

In the paper by Y. Celik (Karamanoglu Mehmetbey University) and E. Ulker (Selcuk University), their research proposes an improved marriage in honey bees optimization (IMBO) by adding Levy flight algorithm for queen mating flight and neighboring for worker drone improving. The IMBO algorithm's performance and its success are tested on the well-known six unconstrained test functions and compared with other metaheuristic optimization algorithms.

M. Baygin (Ardahan University) and M. Karakose (Fırat University) study a new approach of immune system-based optimal estimate for dynamic control of group elevator systems. The method is mainly based on estimation of optimal way by optimizing all calls with genetic, immune system and DNA computing algorithms, and it is evaluated with a fuzzy system. With dynamic and adaptive control approach in this study, a significant progress on group elevator control systems has been achieved in terms of time and energy efficiency according to traditional methods.

The paper by M. Karakose (Fırat University) proposes a reinforcement-learning based artificial immune classifier. The proposed new approach has many contributions according to other methods in the literature such as effectiveness, less memory cell, high accuracy, speed, and data adaptability. Some benchmark data and remote image data are used for experimental results. The comparative results with supervised/unsupervised based artificial immune system, negative selection classifier, and resource limited artificial immune classifier are given to demonstrate the effectiveness of the proposed new method.

In their paper, T. J. Choi et al. (Sungkyunkwan University) and (Daegu Gyeongbuk Institute of Science and Technology) present an adaptive parameter control DE algorithm. The control parameters of each individual are adapted based on the average of successfully evolved individuals' parameter values using the Cauchy distribution. The experimental results show that their proposed algorithm is more robust than the standard DE algorithm and several state-of-the-art adaptive DE algorithms in solving various unimodal and multimodal problems.

In the paper by R.-J. Ma et al. (Southwest Jiaotong University and CSR Qishuyan Institute Co., Ltd.), the authors present an integral mathematical model and particle swarm optimization (PSO) algorithm based on the life cycle cost (LCC) approach for the heating system planning (HSP) problem. The results show that the improved particle swarm optimization (IPSO) algorithm can more preferably solve the HSP problem than PSO algorithm.

In the paper by M. Tang et al. (National University of Defense Technology and Université Pierre et Marie Curie), they report that the flocking has some negative effects on the human, as the infectious disease H7N9 will easily be transmitted from the denser flocking birds to the human. Their paper focuses on the H7N9 virus transmission in the flocking birds and from the flocking birds to the human. Some interesting results have been shown: (1) only some simple rules could result in an emergence such as the flocking; (2) the minimum distance between birds could affect H7N9 virus transmission in the flocking birds and even affect the virus transmissions from the flocking birds to the human.

Y. Wang et al. (China University of Petroleum) present a memory-based multiagent coevolution algorithm for robust tracking the moving objects. Each agent can remember, retrieve, or forget the appearance of the object through its own memory system by its own experience. Experimental results show that their proposed method can deal with large appearance changes and heavy occlusions when tracking a moving object. 

The paper by Q. Ni and J. Deng (Southeast University and Soochow University) analyzes the performance of PSO with the proposed random topologies and explores the relationship between population topology and the performance of PSO from the perspective of graph theory characteristics in population topologies. Further, in a relatively new PSO variant which named logistic dynamic particle optimization, an extensive simulation study is presented to discuss the effectiveness of the random topology and the design strategies of population topology. 

Y. Zhou and H. Zheng (Guangxi University for Nationalities, Guangxi Key Laboratory of Hybrid Computation and IC Design Analysis) propose a novel complex valued cuckoo search algorithm. They use complex-valued encoding to expand the information of nest individuals and denote the gene of individuals by plurality. The value of independent variables for objective function is determined by modules, and a sign of them is determined by angles. The position of nest is divided into real part gene and imaginary gene. Six typical functions are tested, and the usefulness of the proposed algorithm is verified.

The paper by R. Alwee et al. (Universiti Teknologi Malaysia) introduces a hybrid model that combines support vector regression (SVR) and autoregressive integrated moving average (ARIMA) to be applied in crime rates forecasting. Particle swarm optimization is used to estimate the parameters of the SVR and ARIMA models. The experimental results show that their proposed hybrid model is able to produce more accurate forecasting results as compared to the individual models.

Finally, K. S. Lim et al. (Universiti Teknologi Malaysia, Universiti Malaysia Pahang, and University of Malaya) describe an improved Vector Evaluated Particle Swarm Optimization algorithm by incorporating the nondominated solutions as the guidance for a swarm rather than using the best solution from another swarm. The results suggest that the improved Vector Evaluated Particle Swarm Optimization algorithm has impressive performance compared with the conventional Vector Evaluated Particle Swarm Optimization algorithm.

